# Anemia and associated factors among pregnant women attending antenatal care in public health facilities of Garowe City, Puntland, Somalia

**DOI:** 10.1371/journal.pone.0354666

**Published:** 2026-07-24

**Authors:** Abdirisak Nur Yusuf, Adisu Birhanu, Alemayehu Tesfaye, Birhanu Shegene, Obsan Kassa, Tadesse Gure Eticha, Dawit Firdisa, Berhe Gebremichael

**Affiliations:** 1 School of Public Health, College of Health and Medical Sciences, Haramaya University, Harar, Ethiopia; 2 Center for Postgraduate Studies, University of Bosaso, Garowe, Somalia; 3 School of Medicine, College of Health and Medical Sciences, Haramaya University, Harar, Ethiopia; Public Library of Science, UNITED KINGDOM OF GREAT BRITAIN AND NORTHERN IRELAND

## Abstract

**Background:**

Anemia is a prevalent global public health issue, especially among pregnant women in developing countries. Limited research exists on anemia in pregnant women in Somalia, particularly in Garowe City. This study aims to determine the prevalence of anemia and its associated factors among pregnant women attending antenatal care in public health facilities in Garowe City, Puntland, Somalia, from December 1, 2023, to January 1, 2024.

**Methods:**

An institution-based cross-sectional study was conducted with 422 randomly selected pregnant women in public health facilities. Data were collected using EpiData version 4.6 and analyzed with SPSS version 25. Bivariate and multivariable logistic regression analyses identified factors associated with anemia, with statistical significance set at P < 0.05.

**Results:**

The prevalence of anemia among pregnant women was 38.6% (95% CI: 33.9%, 43.6%). Significant associated factors included: rural residence (AOR = 2.58, 95% CI: 1.18–5.64), multigravidity (AOR = 2.01, 95% CI: 1.03–3.89), lack of nutritional counseling (AOR = 1.65, 95% CI: 1.09–2.52), positive malaria test (AOR = 3.58, 95% CI: 1.08–11.98), and not consuming one extra meal (AOR = 2.19, 95% CI: 1.15–4.19).

**Conclusions:**

Anemia in pregnant women in Garowe City is a moderate public health concern according to WHO standards. Factors such as residence, gravidity, nutritional counseling, malaria status, and dietary habits significantly correlate with anemia. Public health measures should focus on enhancing nutritional counseling, promoting extra meal consumption, and preventing malaria during pregnancy.

## Introduction

Anemia is characterized by a reduction in the concentration of red blood cells in circulation or a decrease in hemoglobin/hematocrit concentration, leading to an impaired capacity to transport oxygen. It measures the ratio of red cell concentration to blood volume, which is traditionally used to quantify the decline [1]. According to the WHO definition, hemoglobin (Hb) <11 g/dL or a hematocrit <33% at any time during pregnancy is considered anemia of pregnancy. It is classified as mild (10.0–10.9 g/dL), moderate (7.0–9.9 g/dL), and severe (lower than 7.0 g/dL) anemia based on the level of hemoglobin concentration [2]. Iron deficiency can be diagnosed by serum ferritin-level measurement, with a threshold value of <30 μg/L. Routine hemoglobin testing, at least once per trimester, and blood ferritin-level testing in the first trimester are the recommended methods for screening for anemia caused by iron deficiency in all pregnant women [3]. The recommended daily iron intake for pregnant women is 27 mg, and proper nutrition can help prevent iron deficiency anemia during pregnancy. Dietary sources of iron include fish, chicken, and lean red meat, as well as dry beans, peas, dark green leafy vegetables, and iron-enriched breakfast cereals [4].

Anemia is a serious global public health issue that is responsible for significant morbidity and mortality, particularly in women of reproductive age (WRA) living in less developed countries [5]. It is estimated to affect about one-third of WRA worldwide, in which the burden is considerably greater for pregnant women [6]. Evidence shows that 293.1 million (around 43%) of pregnant women are anemic worldwide, particularly in low- and middle-income countries (LMICs) where prevalence can reach up to 60% [7–9]. Globally, iron deficiency approximately 841,000 deaths and 35,057,000 disability-adjusted life years lost [10].

Anemia causes many complications and has been related to reduce work capacity, reduced ability to execute activities of daily living, reduced cognitive function, and fatigue, among others [11]. Anemic pregnant women will be at risk of low physical activity and increased maternal mortality and morbidity, especially those with severe anemia [12]. Both the mother and the fetus may suffer from severe anemia. Poor pregnancy outcomes are attributed to anemia when hemoglobin levels are below 6 grams per deciliter. Severe maternal anemia can lead to low birth weight, fetal deaths, spontaneous abortions, and premature birth [13].

Global initiatives such as Sustainable Development Goals (SDGs), the Global Alliance for Improved Nutrition (GAIN), and the Anemia Action Alliance aim to reduce anemia prevalence by promoting nutritional interventions, improving healthcare access, and fostering education. The SDGs aim to end all forms of malnutrition by 2030, which includes reducing anemia in pregnant women [14]. Anemia is a setback to achieving SDG targets, particularly in LMICs, where young women face higher anemia prevalence due to socio-economic factors [14]. GAIN works to improve nutrition outcomes by enhancing the availability and consumption of nutritious foods, which is essential in preventing anemia [15]. The alliance supports interventions that increase awareness and education about nutrition among pregnant women, addressing key risk factors such as dietary practices and food consumption [15]. This alliance focuses on reducing anemia through coordinated global efforts, emphasizing the importance of iron supplementation and healthcare access [16]. It advocates for policies that address the multifactorial causes of anemia, including infections like malaria and socio-economic determinants [16].

Anemia is particularly prevalent in developing nations due to a combination of factors, primarily iron deficiency, along with inherited conditions like thalassemia and acute and chronic infections that cause inflammation and blood loss; additionally, deficiencies in essential vitamins and minerals, such as folate, vitamin B12, and vitamin A, contribute to the issue. Sociocultural factors further exacerbate the situation, including poverty, ignorance, poor eating habits, parasitic infestations, blood loss, tuberculosis, malaria, early pregnancies, high parity, short inter-pregnancy intervals, cultural beliefs and practices, non-use of insecticide-treated bed nets, and delays in seeking prenatal care. As a result, anemia in pregnant women remains one of the most challenging public health issues in developing nations [17,18].

No published evidence addresses the prevalence and associated factors of anemia among pregnant women in the northeastern part of Somalia, specifically Garowe City. Therefore, this study aimed to determine the prevalence of anemia and its associated factors for pregnant women in Garowe City, Puntland, Somalia. The findings from this study underscore the necessity for targeted public health policies promoting iron supplementation and nutritional education, guiding policymakers in resource allocation to combat anemia, while also emphasizing the importance of routine screening and tailored interventions by healthcare providers to enhance maternal health outcomes. Ultimately, it contributes to improving maternal and neonatal health in the region.

## Materials and methods

### Study design and setting

An institution-based cross-sectional study was conducted among pregnant women attending antenatal care in Garowe City, Puntland, Somalia, in public health facilities, from December 1, 2023, to January 1, 2024. Puntland is located in the northeastern region of Somalia. Garowe City is the capital city of the Puntland state of Somalia and is situated in the Nugal region. Garowe City is the third largest city in Puntland and has an estimated total population of 70.000 [19]. Garowe City has non-governmental organizations like UNFPA, UNICEF, USAID, UNHCR, UNDP, UNHABITAT, FAO, and others. Garowe city has one public referral hospital, four private hospitals, and six public health centers. The study was carried out in public health facilities that provide health services to over one million people, with six primary departments [20].

### Population and sampling

All pregnant women who visited the public health facilities of Garowe City for antenatal care follow-up were the source population. In contrast, pregnant women who visited the public health facilities in Garowe city during the study period were the study population. Pregnant women who were unable to provide appropriate information (those who had a severe illness during the study period and severe disability) were excluded from the study.

The sample size was determined using both a single and a double population proportion formula. The maximum sample size was achieved by using a single proportion formula by considering a confidence level of 95% and a margin of error of 5%, and a 53.1% prevalence of anemia among pregnant women from a previous study conducted in Jowhar District, Somalia [21]. By adding a 10% non-response rate, the final sample size for the study was calculated to be 422.

A total of three public hospitals and six health centers are available in the city of Garowe. Among them, four health institutions were randomly selected by the lottery method. Proportional allocation was used to select study participants from the selected hospitals and health centers using the last 6 months of registration data of the selected health institutions based on the number of patients flows or visits every month (the total number of pregnant women who visited Garowe General Hospital, Gol Udug General Hospital, Jowle, and Jilab Public Health Centers per month was 600, 400, 450, and 335, respectively). Therefore, the required sample sizes from Garowe General Hospital = 142, Gol Udug Hospital = 95, Jowle Health Center = 106, and Jilab Public Health Center = 79. Using the previous registration data of selected hospitals and health centers as a sampling frame, the study participants were selected using a systematic random sampling technique until the total sample size was reached, using a kth interval, by dividing the total population in selected hospitals for the required sample size, which was 4.

### Data collection and quality control

Structured questionnaires were used to collect data using face-to-face interview techniques to obtain data on socio-demographic, history of chronic disease, history of infectious disease, obstetric factors, and other clinical conditions. The questionnaire was initially developed in the English language and then translated into the local language, Somali, for better understanding by the data collectors and respondents. Two diploma public health and two diploma clinical nurses were recruited as data collectors, and one bachelor of medical laboratory was recruited as a supervisor. Two days of training were given for data collectors and a supervisor on how to approach and recruit study participants, interview techniques, and how to fill out the questionnaires. Then, the data collectors and supervisor conducted pre-testing on 5% of the total sample size outside the study area at the Jawle Health Center before the actual data collection began in the city of Garowe, and necessary corrections were made. Hemoglobin was recorded from their cards/charts since it is a routine investigation for pregnant women during ANC follow-up. Results of blood film for malaria and stool examination were also recorded from the charts.

### Operational definitions

#### Anemia.

Anemia in pregnancy is defined as a condition characterized by low hemoglobin levels, specifically below 11 g/dl according to the World Health Organization (WHO). It is classified into three categories based on hemoglobin concentration: mild (9–10.9 g/dl), moderate (7–8.9 g/dl), and severe (<7 g/dl) [22,23]. We used the Hb level component of the complete blood count recorded in the women’s medical registration.

#### Gravidity.

Refers to the number of times a woman has been pregnant, encompassing all pregnancies regardless of the outcome. It is categorized into three classifications: primigravida, multigravida, and grand multigravida. Primigravida denotes a woman who is pregnant for the first time, while multigravida refers to those who have had two or more pregnancies. A grand multigravida is used for women who have experienced five or more pregnancies [24–26].

#### Malaria test.

Malaria tests were conducted using microscopic blood films, and results were classified as positive or negative based on the result registered in the medical registration.

### Data processing and analysis

The collected data were checked for completeness, coded, and double-entered into Epi Data version 4.6 to check for inconsistency, and then exported to Statistical Package for Social Science (SPSS) version 25 software for analysis. Descriptive analysis was done and presented in tables and charts. Bivariate and multivariable logistic regression were done to identify factors of association and their degree of association with anemia. Variables with a p-value < 0.25 in bivariable regression were then included in multivariable analysis. Multicollinearity was checked by the variance inflation factor (variance inflation factors >10 and standard error >2 were considered suggestive of the existence of multicollinearity), and model goodness-of-fit was checked using the Hosmer-Lemeshow test (P = 0.67). Finally, the level of statistical significance was declared at P-value < 0.05 with an adjusted odds ratio of 95% confidence intervals.

### Ethics approval and informed consent

Ethical clearance was obtained from Haramaya University, College of Health and Medical Sciences, Institutional Health Research Ethics Review Committee (IHRERC) with reference number IHRERC/210/2023. A letter of permission was obtained from the district administrative health bureau. The purpose and benefit of the study were explained to the guardians/parents and the study participants before data collection. Informed, voluntary, written, and signed consent was taken from each head of the health facility and the participants. Confidentiality was maintained, and any information and findings obtained during the study were kept confidential.

## Results

### Socio-demographic and economic characteristics

The study included 422 pregnant women in total, resulting in a 100% response rate. The mean age of the pregnant women was 28.68 (SD ± 5.6) years, with a range of 18–45 years. Two hundred seventy-seven (65.6%) of the pregnant women were within the age group of 25–34 years. Regarding the place of residence, the majority, 389 (92.2%), of the pregnant women were urban dwellers, and almost all, 413 (97.9%), were Muslims. Ethnically, 386 (91.5%) were Somalis. More than two-thirds, 288 (68.2%), were housewives. Regarding the educational status of pregnant women, 263 (63.2%) of them had no formal education. Two hundred seventy-one (64.1%) of the participants’ families earn a monthly income of <$300 (Table 1).

**Table 1:** Socio-demographic and economic characteristics of pregnant women who visited public health facilities in Garowe City, Puntland, Somalia, 2024 (n = 422).

Variables	Categories	Frequencies (n)	Percentage (%)
Age	15-24	13	3.1
	25-34	277	65.6
	>35	132	31.3
Residence	Urban	389	92.2
	Rural	33	7.8
Marital status	Married	388	91.9
	Divorced	30	7.1
	widowed	4	.9
Religion	Muslim	413	97.9
	Christian	9	2.1
Ethnicity	Somali	386	91.5
	Somali bantus	22	5.2
	Arab	6	1.4
	Oromo	8	1.9
Occupation	Government employed	8	1.9
	Private organizational employee	29	6.9
	Daily laborer	86	20.4
	House wife	288	68.2
	Others	11	2.6
Educational Level	Unable to read and write	98	23.2
	Able to read and write	165	39.1
	Primary	136	32.2
	Secondary	18	4.3
	College and above	5	1.2
Monthly Income (in U.S dollars)	300-1000	271	64.1
	200-299	84	19.9
	100-199	58	13.7
	0-99	9	2.1

### Reproductive health and health service utilization

According to the study, the mean gravidity was 2.7 (SD = 1.14), and 141 (33.4%) of the pregnant women were multigravida. Three hundred thirty (78.2%) of the pregnant women had only one ANC visit, and 210 (49.8%) women received health and nutrition education during their current pregnancy. Furthermore, 184 (43.6%) women received iron/folic acid supplementation during their ANC visits (Table 2).

**Table 2:** Reproductive health and Healthcare service utilization at public health facilities in Garowe City, Puntland, Somalia, 2024 (n = 422).

Variables	Categories	Frequencies	Percentage (%)
Gravidity	Primigravida	55	13
	Multigravida	331	78.4
	Grand multigravida	32	7.6
History of abortion	Yes	35	8.3
	No	387	91.7
Frequency of attending ANC visits	1	330	78.2
	2	70	16.6
	3	18	4.3
	4 and above	4	0.9
Health/nutrition education	Yes	210	49.8
	No	212	50.2
Iron-folic acid supplementation	Yes	184	43.6
	No	238	56.4

### Acute and chronic medical conditions

In this study, 45 (10.7%) women were sick in the two weeks before the study, 28 (6.6%) had hypertension, and 20 (4.7%) had diabetes mellitus. In terms of stool examination, parasites were seen in 25 (5.9%) of the pregnant women’s stool samples. Regarding blood film examination, 16 (3.8%) of the women were positive for malaria (Table 3).

**Table 3:** Acute and chronic medical conditions at public health facilities in Garowe City, Puntland, Somalia, 2024 (n = 422).

Variables	Categories	Frequencies	Percentage (%)	
Sickness in the two weeks before the study	Yes	45	10.7	
	No	377	89.3	
Type of sickness (n = 45)	Diarrhea	18	4.3	
	Vomiting	14	3.3	
	Cough	7	1.7	
	Fever	6	1.4	
Hypertension	Yes	28	6.6	
	No	394	93.4	
Diabetes mellitus	Yes	20	4.7	
	No	402	95.3	
Type of diabetes mellitus (n = 20)	Type 1	1	0.2	
	Gestational diabetic	19	4.5	
Any other diagnosed chronic illness	Yes	3	0.7	
	No	419	99.3	
Stool examination results	No parasite seen	397	94.1	
	Parasite seen	25	5.9	
Type of parasite seen (n=25)	Entamoeba histolytica	12	2.8	
	Giardia lamblia	13	3.1	
Blood film examination for malaria	Negative	406	96.2	
	Positive	16	3.8	

### Prevalence of anemia among pregnant women

**Fig 1:**
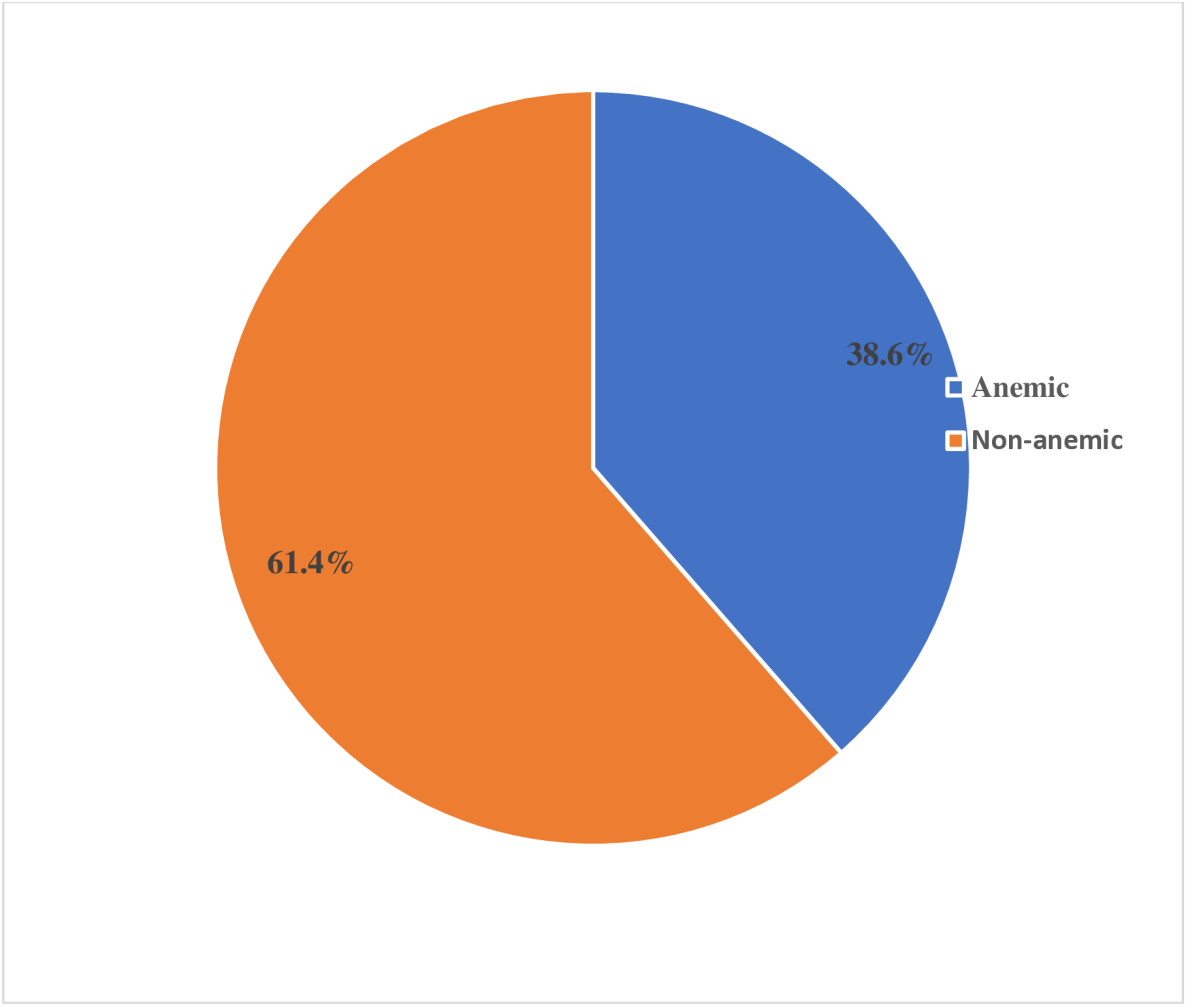
Prevalence of anemia among pregnant women in Garowe City, Puntland, Somalia, 2024.

**Fig 2:**
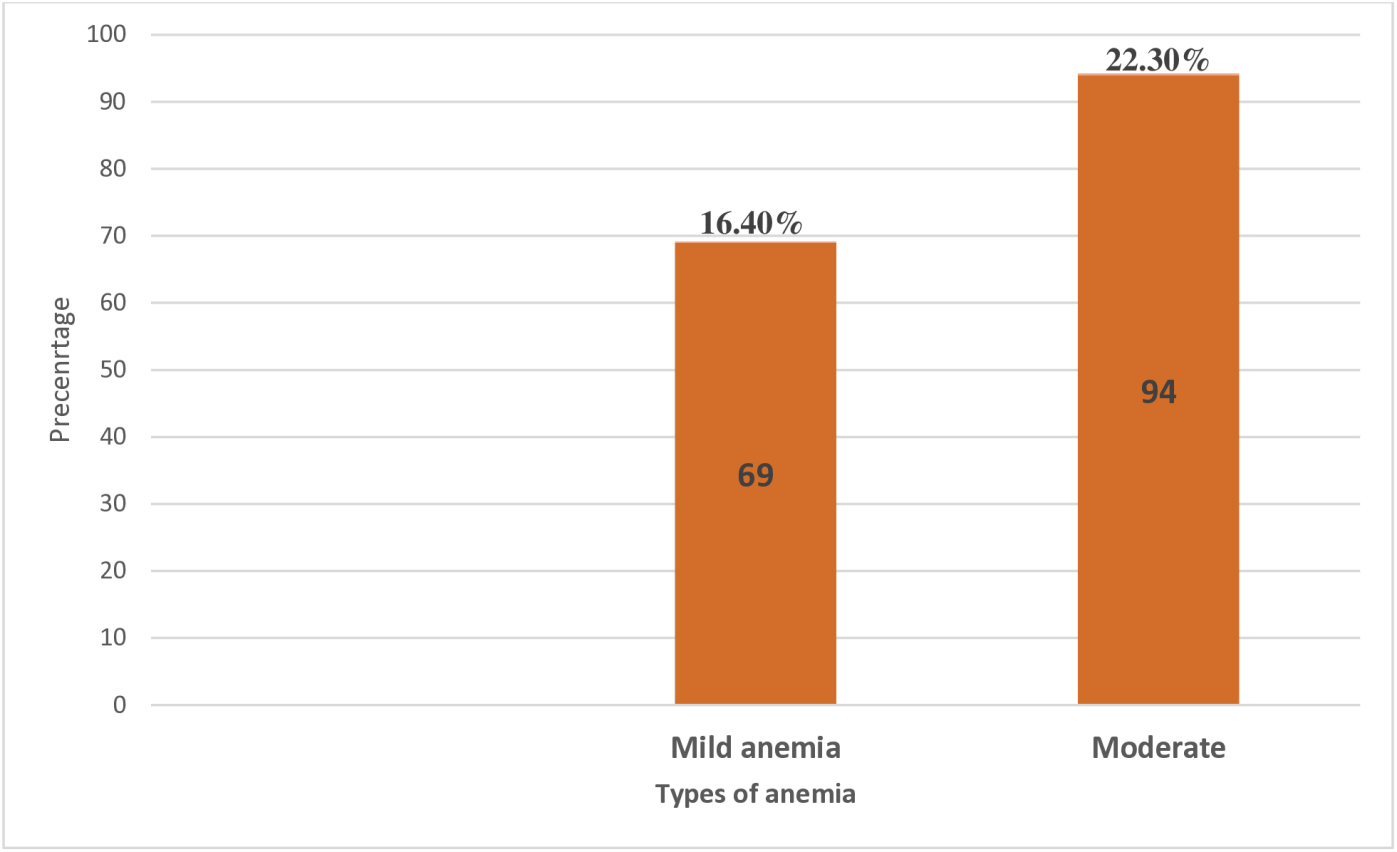
Types of anemia among pregnant Women attending Public Health Facilities in Garowe City, Puntland, Somalia, 2024.

The overall prevalence of anemia in this study was 38.6% (95% CI: 33.9%−43.6%) **(**Fig 1). This prevalence varied among study participants and was reported as mild anemia (16.4%) and moderate anemia (22.3%) (Fig 2). Among pregnant women aged 25–34 years, the prevalence of anemia was 24.2%, and it was 30.9% among multiparous pregnant women.

### Factors associated with anemia among pregnant women

In this study, various factors were included in the logistic regression analysis to identify factors independently associated with anemia in pregnancy. Accordingly, variables such as maternal age, residence, educational level, gravidity, history of abortion, nutritional counseling, history of hypertension, malaria blood film test status, presence of parasite in the stool, and meal frequency were included in the binary logistic regression analysis. All independent predictors having a p-value < 0.25 in the bivariable analysis were considered for the final model of multivariable logistic regression analysis. Accordingly, independent variables like residence, gravidity, history of nutritional counseling, positive malaria test, and utilization of extra meals remained significantly associated with anemia among pregnant women.

Pregnant women from rural areas were 2.6 times more likely to develop anemia compared to urban dwellers [AOR = 2.58; 95% CI (1.18–5.64)]. The odds of having anemia were two times higher among multigravida women than primigravida mothers [AOR = 2.01; 95% CI (1.03–3.89)]. Similarly, receiving no nutritional counseling increased the odds of developing anemia [AOR = 1.65(1.09–2.52)]. Furthermore, the likelihood of anemia was nearly 3.6 times higher among pregnant women who were positive for malaria than those with negative results [AOR = 3.58(1.08–11.98)]. Finally, consuming no extra iron-containing meals during pregnancy was significantly associated with anemia. Thus, women who did not consume an extra meal during pregnancy were nearly three times more likely to develop anemia compared to their counterparts [AOR = 2.62 (1.15–5.95)] (**Table 4**).

**Table 4:** Bivariable and Multivariable logistic regression analysis of factors associated with anemia among pregnant women in Garowe City, Puntland, Somalia, 2024.

Characteristics	Categories	Anemia status		COR (95%CI	AOR (95%CI)
		Anemic (%)	Non-anemic (%)		
Age (years)	15-24	7(4.3)	6(2.3)	1	1
	25-34	102(62.6))	175(67.6)	0.5(0.16-1.53))	0.49(0.15-1.57)
	>35	54(33.1)	78(30.1)	0.59(0.19-1.86)	0.69(0.21-2.31)
Residence	Urban	142(87.1)	247(95.4)	1	1
	Rural	21(12.9)	12(4.6)	3.1(1.45-6.37)	2.58(1.18-5.64) *
Gravidity	Primigravida	15(9.2)	42(16.2)	1	1
	Multigravida	148(90.8)	217(83.8)	1.91(1.02-3.57)	2.01(1.03-3.89) *
History of abortion	Yes	18(11.0)	17(6.6)	1.77(0.88-3.54)	1.38(0.65-2.91)
	No	145(89.0)	242(93.4)	1	1
Counseling on nutrition	Yes	60(36.8)	132(51.0)	1	1
	No	103(63.2)	127(49.0)	1.78(1.20-2.66)	1.66(1.09-2.52) *
History of Hypertension	Yes	15(9.2)	13(5.0)	1.92(0.89-4.14)	1.24(0.52-2.93)
	No	148(90.8)	246(95.0)	1	1
Blood film test status for malaria	Negative	255(98.5)	254(98.1)	1	1
	Positive	12(7.4)	4(1.5)	5.07(1.61-16.0)	3.58(1.08-11.98) *
Stool exam for parasites	No parasite seen	151(92.6)	246(95.0)	1	1
	Parasite seen	12(7.4)	13(5.0)	1.50(0.67-3.38)	0.98(0.40-2.41)
Utilize Extra meals	No extra meal	17(10.4)	11(4.2)	2.63(1.19-5.76)	2.62(1.15-5.95) *
	Has an extra meal	146(89.6)	248(95.8)	1	1

Keywords: 1=Reference, COR=Crude Odds Ratio, AOR= Adjusted Odds Ratio, CI=Confidence Interval, P-value ≤ 0.05=*, P-value ≤ 0.01= **.

## Discussion

The results of this study showed that 38.6% (95% CI: 33.9%−43.6%) of people had anemia. According to the current study, anemia is classified as a moderate public health issue among pregnant women in the study area based on the WHO cut-off points for the public health significance level of anemia [27].

The prevalence of anemia in this study is accurately consistent with a global meta-analytical study, with a result of 38.6% [28]. It is also in line with a study done in the Southwest part of Ethiopia (38.20%) [29] and another study done in Ethiopia (39.94%) [30]. However, this study’s findings are by far lower than other studies in the Boditti health center, with a prevalence of 60% [31], in Kenya (57%) [32], and in Ghana(50.8%) [33]. The possible justifications might be due to differences in the geographical setting of the study population, differences in the sample size of the study, socioeconomic status, and methods of assessment. On the other hand, the finding of this study (38.6%) is higher than the prevalence of studies done in Ethiopia (24.1%) [34], southeast of Ethiopia (27.9%) [35], northeast of Ethiopia (24.2%) [36], and in Tikur Ambessa Specialized Hospital in Addis Ababa (23.1%) [37]. The possible justification for these discrepancies can be explained by the time gap between study periods. Other possible reasons might be due to differences in dietary habits, health-seeking behaviors, and different lifestyles of the community, which might be better in the current study population. Another possible explanation might be that currently, the government is motivating communities towards health services, including pregnant women.

The current study revealed that maternal residence was found to be an independent predictor of maternal anemia in pregnancy. Thus, pregnant women from rural areas were 2.34 times more likely to encounter anemia in pregnancy compared to their counterparts (urban dwellers). This result is also supported by previous studies conducted elsewhere, such as studies from eastern Ethiopia [38,39], in which a higher proportion of anemia was reported in women from rural settings. This may be due to a higher burden of anemia in rural areas since they are less likely to have access to health care services than urban dwellers. Moreover, urban women have more exposure to information about health and nutrition, which enables them to seek better health care services and pregnancy outcomes since they have access to media that could allow mothers to have better choices and decisions for their health as well as for their unborn fetus.

Maternal gravidity was also independently associated with anemia in pregnant women. Accordingly, multigravida women had a greater likelihood of developing anemia than the primigravida ones, which is in line with another study done in Ethiopia [40]. This might be explained due to increased demand, depleted iron stores, and maternal red cell expansion in different levels of gravidity and trimesters of previous pregnancies.

Furthermore, one of the major factors influencing anemia during the current pregnancy was the absence of dietary guidance during pregnancy. This result is consistent with another study that found nutritional advice to be an independent predictor of anemia in Dassie Town [41], where nutritional counseling was an independent predictor of anemia. The reason for this could be that nutritional counseling helps women eat more, improves their nutritional status, and ultimately lowers their chance of becoming anemic.

The current study also found that pregnant women diagnosed with malaria were more likely to be anemic compared to those who were not infected with malaria. Sequestration of malaria parasites in the placenta avoids splenic clearance; thus, it makes pregnant women susceptible to malaria [42]. Maternal anemia and low birth weight can be caused by malaria in several ways, such as the immune system destroying parasitized red blood cells, excessively removing non-parasitized erythrocytes, and impaired erythropoiesis due to bone marrow dysfunction [43]. This finding was in line with studies conducted in Ethiopia [44] and Indonesia [45].

This study is not without limitations. It is difficult to establish a temporal relationship between the independent and dependent variables due to the nature of the cross-sectional study. Since we used secondary data, the laboratory results so we had no control over the laboratory procedures that may lead to measurement bias. Additionally, hemoglobin level has to be adjusted for smokers and altitude, but we do not have information in this regard. Furthermore, potential variables like food security, iron supplementation adherence, gestational age and dietary diversity are missing that should have been adjusted/controlled. Finally, the results could not be generalized to all pregnant women as the setting is limited to health facility-based, and also, many women might not attend ANC.

## Conclusions

This study indicated that nearly two-fifths of pregnant women were found to be anemic, indicating anemia as a moderate public health problem among pregnant women in the study area, as per the WHO cut-off value. Residence, gravidity, nutritional counseling, being positive for malaria, and not consuming an extra meal were significantly associated with anemia in pregnancy. Based on the findings, we recommend that district health offices and health institutions in Garowe City should create community awareness and provide counseling for women from rural areas to encourage early antenatal attendance, encourage consumption of extra meals, and prevent malaria during pregnancy. Healthcare providers should also deliver health education to pregnant women during routine care visits regarding how to prevent anemia during pregnancy. This could help promote the healthcare-seeking behavior of individuals, which might be a stepping stone to reducing the prevalence of anemia among pregnant mothers. Finally, future researchers should focus on primary data to reduce the omission of some important variables.

## Supporting information

S1 File Supporting information. (ZIP)

## Abbreviations

ACD: Anemia of Chronic Disorders
HB: Hemoglobin
IDA: Iron Deficiency Anemia
MCV: Mean Corpuscular Volume
RBC: Red Blood Cells
SPSS: Statistical Package for Social Sciences
VAD: Vitamin A Deficiency
VB12: Vitamin B 12
WBC: White Blood Cells

## References

[1] Normochromic normocytic anemia. www.ncbi.nlm.nih.gov 2023;10(3):4–9. 33351438

[2] Prevalence and factors associated with anemia among pregnant women attending antenatal clinic at St. Paul’s Hospital Millennium Medical College, Addis Ababa, Ethiopia. Hindawi. 2018;4:2301. 10.1155/2018/3942301 PMC6136568 30245724

[3] Diagnosis and treatment of iron-deficiency anaemia in pregnancy and postpartum. PubMed NCBI. 2017;10(3):1007. 10.1007/s00404-017-4526-2 28940095

[4] Iron deficiency in pregnancy. American Journal of Obstetrics and Gynecology. 2020;223(4):516–24. 32184147 10.1016/j.ajog.2020.03.006 PMC7492370

[5] Liu Y, Ren W, Wang S, Xiang M, Zhang S, Zhang F. Global burden of anemia and cause among children under five years 1990-2019: findings from the global burden of disease study 2019. Front Nutr. 2024;11:1474664. doi: 10.3389/fnut.2024.1474664 39474456 PMC11518722

[6] Sunguya BF, Ge Y, Mlunde L, Mpembeni R, Leyna G, Huang J. High burden of anemia among pregnant women in Tanzania: a call to address its determinants. Nutr J. 2021;20(1):65. doi: 10.1186/s12937-021-00726-0 34238307 PMC8268339

[7] Lee AI, Okam MM. Anemia in pregnancy. Hematol Oncol Clin North Am. 2011;25(2):241–59, vii. doi: 10.1016/j.hoc.2011.02.001 21444028

[8] Araujo Costa E, de Paula Ayres-Silva JJA. Global profile of anemia during pregnancy versus country income overview: 19 years estimative (2000–2019). Revista Brasileira de Ginecologia e Obstetrícia. 2023;102(8):2025–31. 10.1007/s00277-023-05279-2 PMC10344983 37233775

[9] Prevalence of anemia and associated factors among pregnant women in north western zone of Tigray, northern Ethiopia: A cross-sectional study. 2015;10(8):1155. 10.1155/2015/165430 PMC4475559 26137321

[10] Stoltzfus RJ. Iron deficiency: global prevalence and consequences. Food Nutr Bull. 2003;24(4 Suppl):S99–103. doi: 10.1177/15648265030244S206 17016951

[11] Tolentino KFJ. An update on anemia in less developed countries. PubMed. 2007;1:44–51. 17620629

[12] LGe 12. al. Anemia and associated factors among pregnant women attending antenatal care clinic in Wolayita Sodo town. National Library of Medicine. 2015;10:155–62. 10.4314/ejhs.v25i2.8 PMC4478267 26124623

[13] Anemia in Pregnancy. NYAS Proceedings. 2006;900(1):125–36.

[14] Merid MW, Chilot D, Alem AZ, Aragaw FM, Asratie MH, Belay DG, et al. An unacceptably high burden of anaemia and it’s predictors among young women (15–24 years) in low and middle income countries; set back to SDG progress. 2023;23(1):1292. 10.1186/s12889-023-16187-5 PMC10321004 37407912

[15] Putra AS, Sulastri D. Nutritional status and anemia in pregnant women: A systematic review. InternatJrnl. 2024;7(5):589–97. doi: 10.33024/minh.v7i5.493

[16] Waye BG, Gurara AM, Awoke KSJW. Prevalence of anemia and associated factor among pregnant women attending ante natal care in Arba Minch public health institutions, South Ethiopia. 2020;5(4):76–83.

[17] Haemoglobin concentrations for the diagnosis of anaemia and assessment of severity. https://appswhoint/ 2013.

[18] al. TE e. Prevalence of Anemia and its Associated Factors among Antenatal Care Attendees in the Public Health Facilities of Pawi District. Journal of Nutritional Medicine and Diet Care. 2020;10:2572–3278.

[19] Tanaka S. United Nations Human Settlements Programme (UN-HABITAT). 2009.

[20] Mohamud SJ. Female gender and conflict resolution in Garowe, Puntland, Somalia. Kampala International University, College of Humanities and Social Sciences. 2016.

[21] Taste SB, Gali N, Tamiru DJAJEC. Magnitude of anemia and its associated factors among pregnant women in Jowhar district, Somalia. 2022;1(3):30–6.

[22] Kaul I, Sunil I, Gupta AJ. Maternal haemoglobin and perinatal outcome in a tertiary care hospital in Jammu city, India. AJIJRCOG. 2017;6(11):5060–6.

[23] Risk factors of anaemia and iron deficiency in Somali children and women. NLM NIH. 2022;18(1):13254. 10.1111/mcn.13254 PMC8710091 34405549

[24] Oladapo-Akinfolarin TT, Akinfolarin OM, Maduagwu MC, Amadi CFJBJI. Effect of gravidity on biochemical parameters in normotensive and hypertensive 3rd trimester pregnant women. 2022;26(2):25–33.

[25] Aragaw F, Mahtemsilllasie M, Jarso HJGO. Grand multiparity and pregnancy related complications among women who gave birth at Jimma University specialized hospital, Jimma, Southwest Ethiopia. 2017;7(4):438.

[26] ACoOa G. Obstetric Data Definitions. The American College of Obstetricians and Gynecologists. 2014.

[27] World health statistics. 2008. www.whoint

[28] Karami M, Chaleshgar M, Salari N, Akbari H, Mohammadi M. Global Prevalence of Anemia in Pregnant Women: A Comprehensive Systematic Review and Meta-Analysis. Matern Child Health J. 2022;26(7):1473–87. doi: 10.1007/s10995-022-03450-1 35608810

[29] Getachew M, Yewhalaw D, Tafess K, Getachew Y, Zeynudin A. Anaemia and associated risk factors among pregnant women in Gilgel Gibe dam area, Southwest Ethiopia. Parasit Vectors. 2012;5:296. doi: 10.1186/1756-3305-5-296 23244514 PMC3533966

[30] Gedefaw L, Ayele A, Asres Y, Mossie A. Anemia and Associated Factors Among Pregnant Women Attending Antenatal Care Clinic in Wolayita Sodo Town, Southern Ethiopia. Ethiop J Health Sci. 2015;25(2):155–62. doi: 10.4314/ejhs.v25i2.8 26124623 PMC4478267

[31] Lelissa D, Yilma M, Shewalem W, Abraha A, Worku M, Ambachew H. Prevalence of anemia among women receiving antenatal care at Boditii Health Center, Southern Ethiopia. Age. 2015;15(19):25.

[32] Okube OT, Mirie W, Odhiambo E, Sabina W, Habtu M. Prevalence and Factors Associated with Anaemia among Pregnant Women Attending Antenatal Clinic in the Second and Third Trimesters at Pumwani Maternity Hospital, Kenya. OJOG. 2016;06(01):16–27. doi: 10.4236/ojog.2016.61003

[33] Wemakor A. Prevalence and determinants of anaemia in pregnant women receiving antenatal care at a tertiary referral hospital in Northern Ghana. BMC Pregnancy Childbirth. 2019;19(1):495. doi: 10.1186/s12884-019-2644-5 31829146 PMC6907326

[34] Kare AP, Gujo AB. Anemia among Pregnant Women Attending Ante Natal Care Clinic in Adare General Hospital, Southern Ethiopia: Prevalence and Associated Factors. Health Serv Insights. 2021;14:1. doi: 10.1177/11786329211036303 34376992 PMC8327009

[35] Kefiyalew F, Zemene E, Asres Y, Gedefaw L. Anemia among pregnant women in Southeast Ethiopia: prevalence, severity and associated risk factors. BMC Res Notes. 2014;7:771. doi: 10.1186/1756-0500-7-771 25362931 PMC4223834

[36] Woldu B, Enawgaw B, Asrie F, Shiferaw E, Getaneh Z, Melku M. Prevalence and Associated Factors of Anemia among Reproductive-Aged Women in Sayint Adjibar Town, Northeast Ethiopia: Community-Based Cross-Sectional Study. Anemia. 2020;2020:8683946. doi: 10.1155/2020/8683946 32832149 PMC7429757

[37] Jufar AH, Zewde T. Prevalence of anemia among pregnant women attending antenatal care at Tikur Anbessa specialized hospital, Addis Ababa Ethiopia. J Hematol Thromb Dis. 2014;2(125):2.

[38] Balis B, Dessie Y, Debella A, Alemu A, Tamiru D, Negash B, et al. Magnitude of Anemia and Its Associated Factors Among Pregnant Women Attending Antenatal Care in Hiwot Fana Specialized University Hospital in Eastern Ethiopia. Front Public Health. 2022;10:867888. doi: 10.3389/fpubh.2022.867888 35719616 PMC9198702

[39] Abdu S, Ali T, Debella A, Assefa N, Teji Roba K. Magnitude and factors associated with anemia among pregnant women admitted to labor ward of Hiwot Fana Specialized University Hospital, Eastern Ethiopia. SAGE Open Med. 2021;9. doi: 10.1177/20503121211047389 34594562 PMC8477710

[40] Getaneh D, Bayeh A, Belay B, Tsehaye T, Mekonnen Z. Assessment of the Prevalence of Anemia and Its Associated Factors among Pregnant Women in Bahir Dar City Administration, North-West Ethiopia. J Preg Child Health. 2018;05(02). doi: 10.4172/2376-127x.1000367

[41] Ahmed S, Hassen K, Wakayo T. A health facility based case-control study on determinants of low birth weight in Dassie town, Northeast Ethiopia: the role of nutritional factors. Nutr J. 2018;17(1):103. doi: 10.1186/s12937-018-0409-z 30400909 PMC6220456

[42] Chua CLL, Khoo SKM, Ong JLE, Ramireddi GK, Yeo TW, Teo A. Malaria in Pregnancy: From Placental Infection to Its Abnormal Development and Damage. Front Microbiol. 2021;12:777343. doi: 10.3389/fmicb.2021.777343 34867919 PMC8636035

[43] Nuru Yesuf N, Agegniche Z. Prevalence and associated factors of anemia among pregnant women attending antenatal care at Felegehiwot Referral Hospital, Bahirdar City: Institutional based cross- sectional study. International Journal of Africa Nursing Sciences. 2021;15:100345. doi: 10.1016/j.ijans.2021.100345

[44] Getahun W, Belachew T, Wolide AD. Burden and associated factors of anemia among pregnant women attending antenatal care in southern Ethiopia: cross sectional study. BMC Res Notes. 2017;10(1):276. doi: 10.1186/s13104-017-2605-x 28705235 PMC5512984

[45] Sumampouw OJ, Nelwan JE, Rumayar AA. Socioeconomic factors associated with diarrhea among under-five children in Manado coastal area, Indonesia. 2019. 10.4103/jgid.jgid_105_18 PMC6906894 31849434

